# Mammography equipment design: impact on radiographers’ practice

**DOI:** 10.1007/s13244-014-0360-2

**Published:** 2014-10-02

**Authors:** Stefanie Costa, Eva Oliveira, Cláudia Reis, Susana Viegas, Florentino Serranheira

**Affiliations:** 1 Department of Science and Technologies of Radiation and Biosignals in Health, Escola Superior de Tecnologia da Saúde de Lisboa (Lisbon School of Health Technology), Lisbon, Portugal; 2 Escola Nacional de Saúde Pública, Universidade Nova de Lisboa, Lisbon, Portugal

**Keywords:** Ergonomics, Digital mammography, Work-related musculoskeletal disorders, WRMSDs

## Abstract

**Objectives:**

Identify radiographers’ postures during frequent mammography procedures related to the mammography equipment and patient characteristics.

**Methods:**

A postural task analysis was performed using images acquired during the simulation of mammography positioning procedures. Simulations included craniocaudal/(CC) and mediolateral-oblique/(MLO) positioning in three different settings: radiographers and patients with similar statures, radiographers smaller than the patients and radiographers taller than the patients. Measurements of postural angles were performed by two raters using adequate software and classified according to the European Standard EN1005-4:2005 + A1:2008.

**Results:**

The simulations revealed that the most awkward posture in mammography is during the positioning of MLO projection in short-stature patients. Postures identified as causing work-related musculoskeletal disorder (WRMSD) risk were neck extension, arms elevated and the back stooped, presenting angles of 87.2, 118.6 and 63.6, respectively. If radiographers were taller than patients, then the trunk and arm postures were not acceptable.

**Conclusions:**

Working in a mammography room leads to awkward postures that can have an impact on radiographers’ health, namely WRMSDs. The results in this study showed that there are non-acceptable postures associated with frequent working procedures in mammography. MLO is the most demanding procedure for radiographer postures and may be related to WRMSDs. Mammography devices should be redesigned considering adjustability for radiographers.

***Main Messages*:**

• *Mammography constraints for radiographers in mammography procedures have not been well studied.*

• *Performing mammography leads to awkward postures that can impact radiographers’ health.*

• *MLO, the most demanding procedure for radiographers, is possibly related to WRMSDs.*

## Introduction

Today’s competitive labour market requires high levels of competitive and physical performance from professionals that can result in stress and fatigue and may promote the occurrence of errors and work-related musculoskeletal disorders (WRMSDs) [1, 2]. The development and adoption of human factors and ergonomic strategies are essential to guarantee safe working conditions during the performance of work tasks, services or when using equipment. These can contribute to a decrease or increase in the quality of a radiographers'  work and their productivity [2, 3].

In mammography departments, radiographers need to adapt their behaviour and performance to the patient’s characteristics when performing mammography procedures [1, 2, 4]. For this reason, it is essential to implement actions focussed on the system (namely the equipment) and in the examination room (layout) so that working conditions can be improved and radiographers' health risks reduced [1, 4, 5]. Nothing has been done in this area, and we expect to find conflicting mammographic design variables that are difficult for radiographers.

Recently, the Society of Radiographers published a document describing the WRMSDs that can affect radiographers working in mammography rooms. The radiographers indicated that they frequently have to manoeuvre equipment into awkward positions. This, accompanied by time constraints and high workloads, can lead to a range of symptoms (pain, tenderness, swelling and muscle weakness) that often result in WRMSDs (rotator cuff syndrome, tendinitis, trigger finger) [6, 7]. Péniou and Kapitaniak presented methods that allow the analysis of a subject undertaking tasks that can be considered high risk through a drawing of the activity or a picture where the mechanical axis, mass centre and rotation centre are the focus. The vector calculations are performed for each joint, and the results are analysed comparing them with references and standards [8].

The lack of national and international studies on digital mammography (DM) hinders the optimisation of radiographers’ working conditions and performance. It seems assumed that system design is fundamentally guided by mammography equipment specifications in the form of a set of criteria that the final system has to meet, forgetting radiographers’ characteristics. From a patient-safety perspective, it is also possible to consider a range of work system factors contributing to radiographer errors, such as time pressure, verbal or written communication problems, and bad equipment design. Therefore, it is important to explore the gap in the concepts of mammography equipment design.

The objective of this study was to investigate how the design of mammography equipment affects radiographers’ postures during mammography procedures.

## Methods

The study was performed in a private hospital in Lisbon using digital mammography equipment (General Electric Healthcare model Senographe DS).

In a first phase, observation was used to collect data related to the stature of patients who had scheduled a mammography on 3 different days (Monday, Wednesday, Friday) (*n* = 93) and radiographers (i = 7) to identify the extremes (taller and shorter).

Observation was also used to characterise the equipment and the practice, defining the main tasks related to the positioning performed by radiographers during mammography examination on a time scale. The data were collected during three different 6-h shifts on different week days (Monday, Wednesday, Friday) [9].

In a second phase, a simulation of patient positioning for the most frequent mammographic projections [craniocaudal (CC) and mediolateral oblique (MLO)] using volunteers was performed.

Three combinations of radiographer-patient statures were analysed using the data related to the stature extremes:

1. The radiographer was taller than the patient (anthropometric stature radiographer/patient combination: 171 cm/153 cm);
2. The radiographer was shorter than the patient (anthropometric stature radiographer/patient combination: 148 cm/174 cm);
3. The radiographer and patient had approximately the same stature (anthropometric stature radiographer/patient combination: 171 cm/174 cm).

Periods of awkward and prolonged postures during each mammography procedure were analysed from photos obtained during procedure simulations and selected by four raters via consensus (1.245, 1.187 and 1.092 photographs for the three simulations, respectively). For this, a Canon EOS 70D with four frames per second was used. The main body angles (head/neck, trunk and arms) were assessed according to the methodology proposed by Kapitaniac [9] by the same four raters in loco consensus observing the frames. Angle measurements were performed using the Meazure 2.0.158 programme and the postures evaluated and classified according to European Standard BS EN 1005–4: 2005 + A1:2008 (Table 1) on three different levels [10]: acceptable, conditionally acceptable and not acceptable. It was also taken into account whether the radiographer was in a static (no movement) or dynamic posture (performing tasks associated with positioning requiring movement of the joints, namely the shoulder, elbow, knees and neck) [11].

**Table 1:** Reference values for postural assessment (Norma BS EN 1005–4:2005 + A1:2008): Acceptable, conditionally acceptable and not acceptable

Postures	Reference values		
	Acceptable	Conditionally acceptable	Not acceptable
Trunk			
Forward/ backward bending	0°to 20°	20° to 60° or <0°^a,b^	<60°
Sideways bending/ twisting	0° to |10°|		>|10°|
Upper arm			
Flexion abduction	0° to 20°	20° to 60°^c^	>60° or <0°
Head/neck			
Upward/ downward bending	(−)40° to 0°		<−40° or >0°
Sideways bending	0° to |10°|		≈|10°|

^a^Acceptability depends upon the duration of the posture and period of recovery; ^b^acceptable if there is full trunk support; ^c^acceptable if there is full arm support; if there is no full arm support, acceptability depends upon the duration of the posture and period of recovery

Individual interviews were conducted with seven radiographers with experience in DM focussed on physical discomfort, repetition of movements, work sequence and postures [12]. The content analysis technique was used to organise the information analysing the frequency of occurrence of the terms associated with mammography activities and the equipment by three researchers.

During the interview, all radiographers also classified the level of the task demand (effort) according to the Borg CR10 scale at each posture. This scale is used for estimating effort and exertion, breathlessness and fatigue during physical work. The Borg CR10 scale is a category ratio (CR) scale based on the number 10, which represents extreme intensities [10]. Also, it was asked about the equipment characteristics, namely: control station height, accessibility of the monitor, accessibility of compression devices, accessibility of the compression paddles and intensity of the positioning light.

## Results

Patient preparation was identified as the most time-consuming task (informed consent), as was sending the images to the PAC systems at the end of the mammography examination. The time required for C-arm adjustment was variable for the first projection, while the others did not cause a time loss as they were performed automatically by the equipment after the activation of the control button. In MLO views, the average time spent for right breast positioning was slightly higher (13 s) than for the left breast. In addition, it was noticed that the average duration of this task increased gradually when compared to the beginning of the work shift. The duration of breast compression on average reached its highest value in the right breast CC view (14 s) at the end of the shift. The average duration of the breast compression application for MLO views reached its higher value in the middle of the work shift at 12 s. For the time interval prior to effective exposure, a variation in the three periods considered for the study was found, the lowest values always being registered in the middle of the work shift.

### A: Simulation when the radiographer and patient have the same height

#### CC patient positioning

**Fig. 1:**
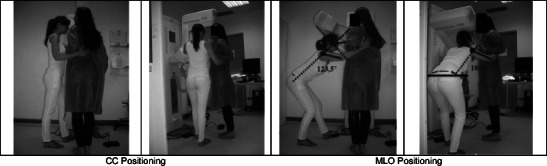
Postures assumed by the radiographer with similar height compared to the patient

The positioning of the breast for CC image acquisition when the radiographer and patient have the same height (Fig. 1 and Table 2) requires the radiographer to assume an orthostatic posture. The spine was aligned with the mid-sagittal plane of the body. The right arm assumes a slight flexion, and the forearm performs a rotation in the inner direction so that the palm of the hand supports the patient's back. The left hand (although not visible in the image) smoothes the breast down and forward with the fingers. The right leg supports part of the radiographer’s body weight, while the left leg performs a slight flexion to reach the compressor foot pedal [1].

**Table 2:** Positioning angles: Radiographer smaller than the patient

Posture	Positioning	Measured angle	Obs.
Trunk			
Forward/ backward bending	CC	0°	Acceptable
	OML	8.1°	Acceptable
Sideways bending/ twisting	CC	12.3°	Not acceptable
	OML	5.8°	Acceptable
Arms			
Flexion	CC	135.5°	Not acceptable
	OML	76.1°	Not acceptable
Abduction	CC	91.6°	Not acceptable
	OML	57.5°	Conditionally acceptable
Head/neck			
Upward/downward bending	CC	(−)10.2°	Acceptable
	OML	20.4°	Not acceptable
Sideways bending	CC	(−)13.2°	Not acceptable
	OML	25°	Not acceptable

According to European Norm 1005–4, the trunk posture is classified as “acceptable” in both static and dynamic body postures once the trunk maintains a vertical posture without inclinations or rotations. The right arm also assumes an “acceptable” posture during the flexion and abduction in dynamic and static contexts. The head/neck did not tilt or rotate during the procedure; therefore, it is always in an “acceptable” position.

#### MLO patient positioning

The radiographer's trunk was flexed and the head/neck extended to observe all breast tissue. The right arm remained flexed, and the hand kept supporting the patient’s back. The radiographer’s body weight was supported by the right leg, which kept a slight flexion, while the left leg was in position to easily reach the compression foot pedal [1]. The trunk position was considered “acceptable” for both static and dynamic situations. On the contrary, the arm position was rated as “not acceptable” in the static situation, whether in flexion or abduction, and was rated as “acceptable” while in motion once this posture was kept for a short duration. The head/neck posture was classified as “not acceptable” in static conditions and “acceptable” in motion.

### B: Simulation when the radiographer is smaller than the patient

**Fig. 2:**
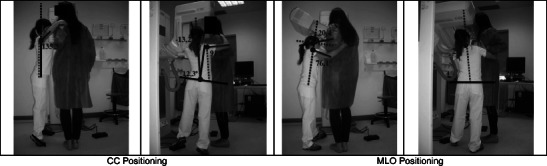
Postures assumed by a radiographer smaller than the patient

If the radiographer is smaller than the patient (Fig. 2 and Table 3), the positioning of the breast for CC patient positioning was done maintaining an orthostatic posture with the vertebral spine aligned to the mid-sagittal plane of the body. Both arms were flexed and abducted. The right forearm revealed internal rotation, and the palm of the hand stayed on the patient’ back, ensuring that the patient remained still and in the correct position. The left hand was positioning the patient’s breast, exerting a slight pressure on it and smoothing it in the anterior direction to ensure the nipple was appropriately positioned before starting the compression. In this specific context, due to the height difference between the radiographer and the patient, the breast area that the radiographer visualised was reduced, showing a tendency for the radiographers to compensate for the height difference by standing on their tiptoes [1]. Both legs remained extended and the left foot placed on the compression foot pedal.

**Table 3:** Positioning angles: Radiographer taller than the patient

Posture	Positioning	Measured angle	Obs.
Trunk			
Forward/ backward bending	CC	6.7°	Acceptable
	OML	63.6°	Not acceptable
Sideways bending/twisting	CC	0°	Acceptable
	OML		
Arms			
Flexion	CC	18.2°	Acceptable
	OML	108.5°	Not acceptable
Abduction	CC	41.4°	Conditionally acceptable
	OML	118.6°	Not acceptable
Head/neck			
Upward/ downward bending	CC	(−)20°	Acceptable
	OML	87.2°	Not acceptable
Sideways bending	CC	Not measurable	
	OML		

The postural assessment of the trunk was classified as “acceptable” either for static posture or in movement. The right arm posture, when in static position, was considered as “not acceptable” in flexion and abduction positions. In movement, its positioning was “acceptable” for both flexion and abduction, following the condition of being maintained for a short period of time. The head/neck postures were “acceptable” for both considered situations.

In MLO patient breast positioning, the radiographer maintained a slight flexion and left lateral inclination of the trunk. The posture of the arms and legs was similar to what was previously described for the CC positioning in this same context. The head/neck stood in extension with a slight left inclination, so the radiographer was able to obtain a good view of all areas under study [1]. According to the European norm EN-1005-4, the trunk posture was “acceptable” for both static and movement situations. In the static situation, the arm was in a “not acceptable” position. This fact compromises the postural acceptability of the body region considered. In a radiographer’s dynamic body situation, the arm flexion posture was “acceptable” once it had been kept for a short period. The arm abduction posture, in the threshold of acceptability, was determined to be “acceptable” according to the same parameters described for the static situation.

The head/neck posture during the static situation was “not acceptable”. On the other hand, when in motion, the posture changes to be “acceptable” if maintained for a short period of time.

### C: Simulation when the radiographer is taller than the patient

**Fig. 3:**
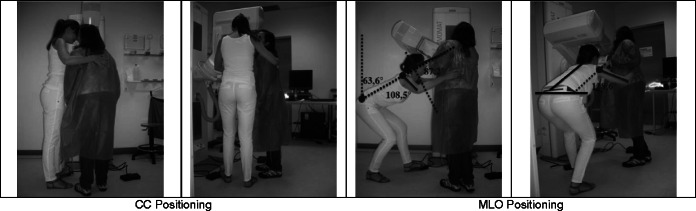
Postures assumed by a radiographer taller than the patient

When the radiographer is taller than the patient (Fig. 3 and Table 4), the positioning of the breast for the acquisition of CC patient positioning requires that the radiographer maintain an orthostatic posture, performing a slight flexion of the trunk and head/neck as a guarantee for the correct visualisation of the breast. Both arms were kept in slight flexion and abduction. The right forearm performed an internal rotation when the radiographer placed an arm around the patient's shoulder. The left hand applied the necessary pressure to hold the breast and to prevent the formation of skin wrinkles. Both legs were aligned with the trunk, with the left in slight flexion to reach the foot pedal and to activate the compression mechanism [1].

**Table 4:** Positioning angles: The radiographer and patient with similar heights

Posture	Positioning	Measured angle	Obs.
Trunk			
Forward/ backward bending	CC	0°	Acceptable
	OML	46.4°	Conditionally acceptable
Sideways bending/ twisting	CC	0°	Acceptable
	OML	0°	Acceptable
Arms			
Flexion	CC	7.3°	Acceptable
	OML	123.5°	Not acceptable
Abduction	CC	31.9°	Conditionally acceptable
	OML	103,5°	Not acceptable
Head/neck			
Upward/ downward bending	CC	(−)14.8°	Acceptable
	OML	51.9°	Not acceptable
Sideways bending	CC	0°	Acceptable
	OML	Not measurable	

According to the European Norm, in CC breast positioning, the trunk assumed an “acceptable” posture for both static and movement situations. Concerning the arm posture, flexion and abduction, the “acceptable” classification was attributed to the small duration of its recovery period. When the radiographer was moving, the arm posture was considered “acceptable” in both positions.

The head/neck posture was “acceptable” for a static posture and also during the radiographer's movement. Side bending or rotations were not identified.

For the MLO patient breast positioning, the radiographer performed severe trunk flexion. The head/neck was in hyperextension to allow the radiographerto have correct visualisation of the breast that was being positioned. Both arms were outstretched above the shoulders and flexed at the elbow level, and while the right hand was resting on the patient’s back, the left hand was positioning the breast on the equipment. The leg, extremely flexed, and the right foot were supporting the majority of the radiographer’s body weight. The left leg was slightly flexed in a way to keep the body balanced and be able to reach the foot pedal [1]. The height difference of both the radiographer and the patient requires greater physical effort from the radiographer to maintain a body position that allows him/her to work (Tables 5 and 6).

**Table 5:** Interview analysis: Work space and postures adopted by the radiographer

Category	Sub-category	Frequency	Percent
Work pace and movements	Repetition of the same movements	7	29.17
	Accelerated pace	4	16.66
	Normal pace	7	29.17
	Depends on the physician's work pace	3	12.5
	Depends on the type of examination	3	12.5
Total		24	100
Adopted postures	Not adequate	8	34.78
	Uncomfortable	5	21.73
	Required postures to perform the examination	2	8.7
	Longstanding	4	17.39
	Corporal adjustment	2	8.7
	Information about this subject does not exist	2	8.7
Total		23	100

**Table 6:** Interview analysis: Situations that trigger physical effort and affected anatomical regions

Category	Sub-category	Frequency	Percent
Situations that trigger physical effort	MLO positioning	10	45.45
	Shorter patients	5	22.72
	Taller patients	3	13.64
	Bigger breasts	2	9.09
	Smaller breasts	1	4.55
	Patient’s 1° mammography	1	4.55
Total		22	100
Affected anatomical regions	Cervical spine	2	14.29
	Shoulder	4	28.57
	Hands	1	7.14
	Lumbar spine	5	35.71
	Legs	2	14.29
Total		14	100

The evaluation of the radiographer’s posture in MLO patient positioning demonstrated that the trunk posture was “not acceptable” in the static position. In motion, the posture happened to be “acceptable” under the condition of being kept for a short period of time. The position of the arms (flexion and abduction) was classified as "not acceptable" in the static situation and "acceptable" in motion for the same reason mentioned in the trunk assessment. The head/neck posture was “acceptable” in both considered situations.

During the interviews, all radiographers referred physical discomfort during the patient positioning to the muscular effort. The positioning of the MLO projection was considered the most demanding by 45.5 % of the radiographers, followed by the positioning of patients who have a shorter stature (22.7 %). The body areas identified as being subjected to greater effort were the shoulder, which is used for positioning the breast (28.6 %) and also the neck (Table 2).

All seven radiographers stated that mammography promotes repetitive movements. Of those, 71 % reported physical fatigue at the end of the work shift (Table 3). Regarding the body postures, radiographers indicated that breast positioning can cause discomfort. As a consequence, radiographers suggested that the implementation of specialised training to correct and adopt the appropriate body postures might reduce the negative impacts on health.

Regarding the equipment, all the radiographers considered that the control station and monitor heights were adequate. The monitor was referred to four times (30.8 %) as being at the level of the radiographers’ eyes.

In the equipment category, 100 % of the radiographers considered the buttons used to adjust the equipment to have an adequate size and location in the gantry. All radiographers evaluated the pedal size as adequate. They stated that the mobility of the pedals allowed an easier positioning of the patient and the breast since their hands were free to work. Their opinion was also unanimous regarding the ease with which the button symbols could be interpreted.

Considering the light field used to position the breast, 71.4 % of the radiographers classified it as sufficient to identify the breast boundaries.

## Discussion

Through this simulation and body posture evaluation according to the European Norms (BS EN 1005–4:2005), it was possible to verify that some of the postures were classified as “not acceptable” mostly for MLO breast positioning considering the static posture of the radiographer. This was also noted by the radiographers who reported physical discomfort required for the positioning of the MLO projections considering the smaller patients.

According to the European Norms, it was possible to verify the postures that had not been classified as “acceptable” in a static position. However, it became “acceptable” when performed during the dynamic context when the lower limbs were fully supported. The body position may be maintained for a short period of time (<2 min) after a recovery period while changing the body posture [2].

Mammography positioning requires a continuous repetition of the same movements during the entire work shift (more than 20 patients per shift). This situation combined with the adoption of “not acceptable” body postures may contribute to an increasing risk to develop WRMSDs [2, 4].

The major risks in breast positioning are caused by the need for continuous observation of the breast while standing too close to the patient and during the breast positioning on the breast support to guarantee that all tissue is included on the image. The first situation requires an exaggerated extension and rotation of the radiographer's neck due to the equipment design and will also have an influence on the adoption of awkward postures at the level of the trunk and legs. In the second situation, the major risks will focus on the arm that is positioning the breast. This positioning requires rotation of the wrist, elbow flexion and shoulder elevation. These movements increase the risk when associated with a constant application of force in the breast to support its weight and to keep it in the proper position performing the compression. Although interventions were not mentioned in this study, they have already been described in other studies, including the existence of different mammography positioning strategies mainly for the MLO projection [1, 4]. The radiographers’ adoption of a lower position relative to the patient, by sitting or kneeling, might result in a lower neck extension and trunk twisting, as well as lesser effort for the lower limbs [1, 4]. The training can also improve the performance of radiographers, minimising awkward positions. However, this focused intervention (worker centred) does not substitute a broader one that minimises constraints such as equipment redesign. Therefore, the mammograph used in this study could not be adjusted by the radiographers, particularly in the MLO position of the breast and when a great discrepancy between the heights of the radiographers and patients existed. It is possible that motion features developed considering only the adjustability for the patient, without considering the radiographers' well-being at work [13, 14]. Mammograph design according to this study does not allow for the comfort and safety of radiographers and patients.

## Conclusions

According to the EN 1005 recommendations, the mammography equipment in use during this simulation was not adjustable for radiographers. CC and MLO mammography proceedings were highly demanding for radiographers, and there are postures classified as “not acceptable” during working procedures in mammography. MLO patient positioning required the radiographers to assume frequent awkward postures, which can increase symptoms, pain and perhaps WRMSDs. This was more evident if there were anthropometric differences between the radiographers and patients. The ergonomic design of the mammography equipment may make a substantive contribution to WRMSD prevention considering radiographers’ anthropometric characteristics. Interventions should have two main approaches: (1) equipment redesign preventing radiographers' awkward postures during mammography positioning; (2) education and training of radiographers in ergonomics and work-related musculoskeletal disorders that include different types of patient and radiographer positioning, for instance, asking the patient to sit when the patient is taller than the radiographer (for CC projection positioning), or the radiographer can sit when the patient is shorter (for MLO projection positioning).

Improving workplace health and safety in radiology and empowering healthcare professionals are means of contributing to hospital quality and patient safety.
